# Effects of low doses of estrone on the proliferation, differentiation and mineralization of osteoprecursor cells

**DOI:** 10.3892/etm.2012.655

**Published:** 2012-08-03

**Authors:** JUN-BEOM PARK

**Affiliations:** Department of Periodontics, Seoul St. Mary’s Hospital, College of Medicine, The Catholic University of Korea, Seoul 137-701, Republic of Korea

**Keywords:** differentiation, estrogen receptor-α, estrogen receptor-β, estrone, osteopontin, osteoblast, proliferation

## Abstract

The purpose of this study was to evaluate the effects of low doses of estrone on the proliferation, differentiation and mineralization of osteoprecursor cells. The effect on cell viability was determined using a 3-[4,5-dimethylthiazol-2-yl]-2,5-diphenyltetrazolium bromide (MTT) assay, whereas differentiation and mineralization were examined using an alkaline phosphatase activity test and alizarin red S staining, respectively. The protein expression of estrogen receptor-α (ER-α), estrogen receptor-β (ER-β) and osteopontin (OPN) is assosciated with bone formation. Cell cultures grown in the presence of estrone at concentrations of 0.1 and 1 nM demonstrated an increase in relative values in the MTT assay and cells grown in the presence of estrone at the 10 nM concentration demonstrated an increase in mineralization. The results of the western blot analysis indicated that the addition of estrone upregulated ER-α and ER-β expression, but downregulated the expression of OPN. Based on these findings, it was hypothesized that a low dose of estrone produces positive effects on the mineralization of osteoprecursor cells. Moreover, these results also suggested that higher doses of estrone may be required to significantly enhance the differentiation and mineralization.

## Introduction

Estrogen has been reported to produce a variety of pleiotropic effects in target tissues as diverse as bone, brain, breast, blood vessel and the male and female gonads (1). It is widely accepted that estrogen plays a significant role in the regulation of bone remodeling and the development and maintenance of the skeleton (2). Estrogen replacement therapy has long been an important therapeutic modality for the prevention and treatment of post-menopausal osteoporosis (3,4). Several types of naturally occurring estrogen have been reported in the literature (5). It has been reported that the most potent naturally occurring estrogen in humans is 17β-estradiol, followed by estrone and estriol (6).

The inhibition of bone resorption by 17β-estradiol is relatively well established (7), but studies investigating the effect of estrogen on osteoblast proliferation and differentiation have produced inconsistent results (8). There is a limited number of studies available with regard to the effects of estrone on the differentiation and mineralization of osteoblasts (3,8). However, the effects of low doses of estrone on bone cells and the underlying mechanisms have not yet been fully investigated.

The present study aimed to examine the effects of various dosages of estrone (0.01 to 10 nM) on the cellular proliferation, differentiation and mineralization of preosteoblasts. Cell viability was evaluated using the 3-[4,5-dimethylthiazol-2-yl]-2,5-diphenyltetrazolium bromide (MTT) assay, and the alkaline phosphatase (ALP) activity test and alizarin red S staining were used to assess the differentiation and mineralization of treated cells, respectively. The expression of proteins associated with bone formation, including estrogen receptor-α (ER-α), estrogen receptor-β (ER-β) and osteopontin (OPN) was evaluated using western blot analysis. To the best of the author’s knowledge, this is the first study to demonstrate the effects of low doses of estrone on the expression of OPN in osteoprecursor cells.

## Materials and methods

### Cell culture

Murine osteoprecursor cells (MC3T3-E1 cells) were grown in α-minimum essential medium (αMEM; Invitrogen, Carlsbad, CA, USA) supplemented with 10% fetal bovine serum (Invitrogen), antibiotics (100 U/ml of penicillin and streptomycin, 100 *µ*g/ml; Invitrogen). The culture medium was changed to osteogenic differentiation medium [(αMEM supplemented with 50 *µ*g/ml ascorbic acid (Sigma, St. Louis, MO, USA) and 10 mM β-glycerophosphate (Sigma)] to induce osteogenic differentiation. The cultures were kept in a humidified atmosphere containing 5% CO_2_ and 95% air at 37°C. Estrone was dissolved in dimethyl sulfoxide (DMSO; Sigma) and filter-sterilized. In order to minimize any differences in cellular growth and differentiation between the controls and treated cultures, an equal amount of DMSO was administered to the controls and treated cultures in each experiment.

### Cellular proliferation

Cells were plated at a density of 1.0×10^4^ cells, 1 ml/well in 12-well plates and the cultures were stimulated with estrone at a range of final concentrations between 0.01 nM and 10 nM. The effects of estrone on the cellular proliferation of the osteoprecursor cells were assessed on day 4. At the end of the incubation time, the MTT reagents were added at a final concentration of 0.5 mg/ml. The cells were incubated for 1 h at 37°C then washed with phosphate-buffered saline (PBS), pH 7.4, followed by the addition of DMSO. Complete dissolution was achieved after gentle agitation. Aliquots of the resulting solutions were transferred into 96-well plates, and absorbance was recorded at 560 and 670 nm using a microplate spectrophotometer system.

### ALP activity assays

Cells were lysed into a buffer which contained 10 mM Tris-HCl, pH 7.4, and 0.2% Triton X-100 and then sonicated for 20 sec at 4°C. Samples were then added to a glycine buffer (100 mM, pH 10.5) containing 10 mM p-nitrophenylphosphate and 1 mM MgCl_2_ and incubated at 37°C in a water bath. Total protein content was determined by comparison with a bovine serum albumin series as an internal standard. The optical density of p-nitrophenol at 405 nm was determined spectrophotometrically and ALP activities were normalized with respect to total protein content.

### Mineralization assay

Cell cultures obtained at day 14 were washed twice with PBS, fixed for 1 h in ice-cold 70% ethanol and then rinsed twice with deionized water. The cultures were stained with 40 mM alizarin red S for 30 min under gentle agitation. To remove non-specifically bound stain, cultures were washed three times with deionized water and once with PBS for 15 min at ambient temperature. To quantify the bound dye, the stain was solubilized by agitation with 10% cetylpyridinium chloride. The absorbance of the solubilized stain was measured at 562 nm.

### Western blot analysis

Osteoprecusor cells were washed twice with ice-cold PBS and solubilized in lysis buffer containing 10 mM Tris-HCl, pH 7.4, and 0.2% Triton X-100. The lysates were centrifuged at 14,000 rpm for 20 min at 4°C to remove the nuclear pellet. The supernatants were boiled in a sodium dodecyl sulfate sample buffer containing β-mercaptoethanol. Equal quantities of the cell extracts were separated using sodium dodecyl sulfate-polyacrylamide gel electrophoresis and transferred onto polyvinylidene fluoride microporous membranes (Immobilon-P membranes; Millipore Corporation, Billerica, MA, USA). Membranes were then blocked for at least 1 h in 0.1% (v/v) Tween-20 in PBS containing 5% (w/v) powdered milk. The membrane was immunoblotted with the desired antibodies which were diluted in the same buffer at the recommended concentrations. The membrane was incubated with horseradish peroxidase-conjugated secondary antibody. The washed blot was developed using enhanced chemiluminescence detection kits.

Mouse antibodies against ER-α, ER-β, OPN and β-actin and the secondary antibodies conjugated to horseradish peroxidase were purchased from Cell Signaling Technology, Inc. (Danvers, MA, USA), Abcam (Cambridge, MA, USA) and Santa Cruz Biotechnology, Inc. (Santa Cruz, CA, USA).

### Statistical analysis

Results are presented as mean ± SD of the experiments and a one-way analysis of variance (ANOVA) was performed to determine the differences between groups using a commercially available program (PASW Statistics 18; SPSS Inc., Chicago, IL, USA). P<0.05 was considered to indicate a statistically significant difference.

## Results

### Cellular proliferation

**Figure 1:**
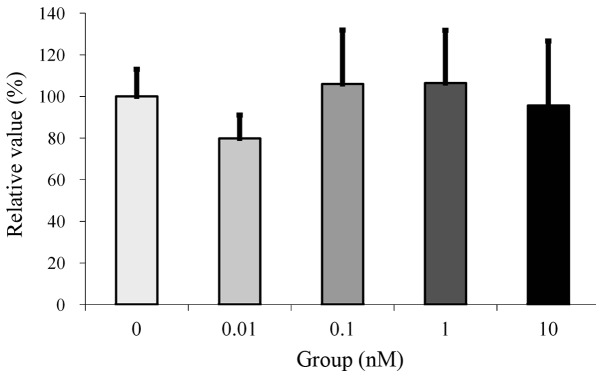
Determination of cellular proliferation using the MTT assay.

Cultures grown in the presence of estrone at the 0.1 and 1 nM concentrations demonstrated an increase in relative values in the MTT assays. However, no significant differences were observed between the groups (Fig. 1).

### ALP assays

**Figure 2:**
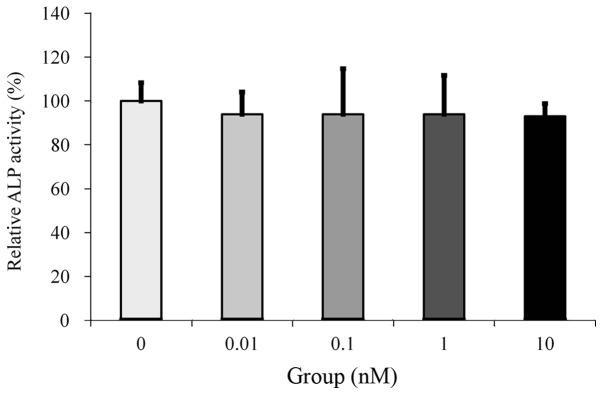
Relative value of ALP activity. ALP, alkaline phosphatase.

Cultures grown in the absence of estrone presented the highest value for the ALP activity, but no significant differences were observed between the groups (Fig. 2).

### Mineralization/calcium deposition assay

**Figure 3:**
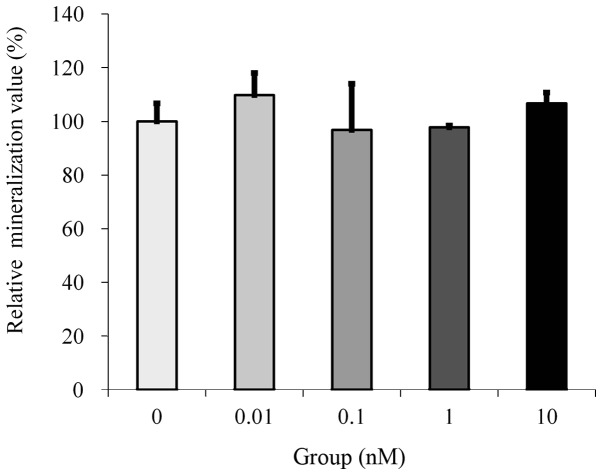
Relative mineralization value following the addition of estrone to the cultures.

Cultures grown in the presence of estrone at the 10 nM concentration demonstrated an increase in mineralization. However, statistically significant differences were not observed between the tested groups (Fig. 3).

### Western blot analysis

**Figure 4:**
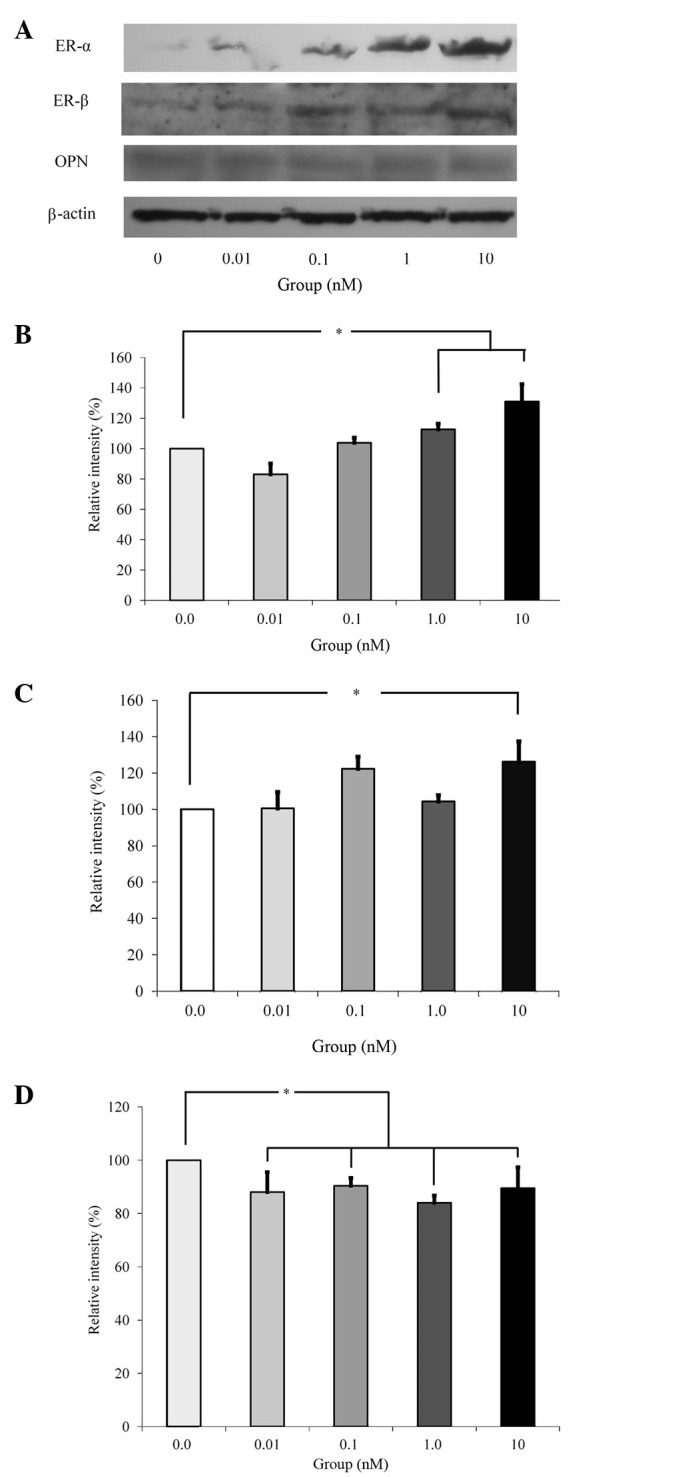
(A) Western blot analysis to detect the expression of the proteins ER-α, ER-β, OPN and β-actin. (B) Quantitative analysis of the expression of ER-α following normalization with β-actin levels by densitometry. ^*^Significant differences compared with the control (non-loaded group; P<0.05). (C) The relative expression of ER-β after normalization with β-actin levels. ^*^Statistically significant increase (P<0.05) compared with the control (non-loaded group). (D) Quantitative analysis of the OPN expression. ^*^Significant differences compared with the control (non-loaded group; P<0.05). ER, estrogen receptor; OPN, osteopontin.

Western blot analysis was performed to detect protein expression following treatment with estrone (Fig. 4A). The results demonstrated that the addition of estrone increased the expression of ER-α and ER-β following normalization with β-actin expression (Fig. 4B and C). Normalization of protein expression revealed that the group treated with 10 nM estrone yielded 131.0±11.5 and 126.3±11.1% of the ER-α and ER-β expression levels compared with the control, respectively. The increase of ER-α and ER-β expression at the 10 nM estrone group was statistically significant (P<0.05). However, estrone appeared to reduce the expression of OPN (Fig. 4D).

## Discussion

In the present study, we examined the effects of low doses of estrone on cell viability and the differentiation and mineralization of osteoblast progenitor cells at predetermined concentrations (0.01 to 10 nM). Additionally, experiments were performed to identify through which pathway the effects of estrone occur.

MTT assays were used in the present study to evaluate cellular proliferation since this assay allows mitochondrial dehydrogenases to oxidize MTT to an insoluble blue formazan product (9,10). The results indicated that although there were increases in the relative values, they were not significant increases, suggesting that cellular proliferation was not affected by the treatment. Previous studies have demonstrated that estrone at a concentration of 10 nM markedly stimulated the proliferation of human breast epithelial HBL-100 cells and that estrone at concentrations of 10 and 100 nM significantly promoted the proliferation and survival of human osteoblastic MG-63 cells (8). This difference in results may be due to the expression of large amounts of ER-α and ER-β, which are responsive to estrogen, in MG-63 cells (11).

Osteoblast differentiation was assessed by ALP activity, which has been reported to be an early marker of osteoblastic cell differentiation (12,13). The results of the present study revealed that estrone demonstrated no significant effects within the range of administered doses. In MG-63 cells, estrone at 100 nM concentration has been demonstrated to markedly increase ALP activity (11). The presence of calcium deposits was evaluated using alizarin red S staining with the aid of cetylpyridinium chloride for quantification (14). Although treatment with estrone at higher concentrations demonstrated a tendency to increase the calcium levels of cellular deposits, this did not reach a statistically significant level. The difference in differentiation and mineralization behavior with regard to the estrone dosage may be attributed to the type of cells, system model, the culturing period or different affinity for ERs (8,15).

Western blot analysis was performed to detect the expression levels of ER-α, ER-β and OPN when cells were treated with estrone. Estrogens and ER modulators bind to ER-α and/or ER-β to form discrete molecular complexes that exert pleiotropic tissue-specific effects by modulating the expression of their target genes (16). The present study demonstrated that estrone influences ER-α, ER-β and OPN expression. OPN is also an important mediator of bone remodeling, and it has been reported to be a negative regulator of calcification (17). Previous studies have revealed that increasing OPN levels through the overexpression of OPN mRNA caused a significant decrease in BMP-2-inducible ALP activity and mineral deposition (17). The western blotting data in the present study may demonstrate the same mechanism.

Based on these findings, it was hypothesized that a low dose of estrone may produce positive effects on the mineralization of osteoprecursor cells. Moreover, these results also suggest that higher doses of estrone may be required to significantly enhance the differentiation and mineralization of these osteoprecursor cells.
